# The Influence of Fatigue, Recovery, and Environmental Factors on the Body Stability of Construction Workers

**DOI:** 10.3390/s24113469

**Published:** 2024-05-28

**Authors:** Daehwi Jo, Hyunsoo Kim

**Affiliations:** Department of Architectural Engineering, Dankook University, 152 Jukjeon-ro, Suji-gu, Yongin-si 16890, Gyeonggi-do, Republic of Korea; highly8755@dankook.ac.kr

**Keywords:** FSTs (falls; slips; and trips), fatigue, body stability, Inertial Measurement Unit (IMU), Dynamic Time Warping (DTW)

## Abstract

In the construction industry, falls, slips, and trips (FST) account for 42.3% of all accidents. The primary cause of FST incidents is directly related to the deterioration of workers’ body stability. To prevent FST-related accidents, it is crucial to understand the interaction between physical fatigue and body stability in construction workers. Therefore, this study investigates the impact of fatigue on body stability in various construction site environments using Dynamic Time Warping (DTW) analysis. We conducted experiments reflecting six different fatigue levels and four environmental conditions. The analysis process involves comparing changes in DTW values derived from acceleration data obtained through wearable sensors across varying fatigue levels and construction environments. The results reveal the following changes in DTW values across different environments and fatigue levels: for non-obstacle, obstacle, water, and oil conditions, DTW values tend to increase as fatigue levels rise. In our experiments, we observed a significant decrease in body stability against external environments starting from fatigue Levels 3 or 4 (30% and 40% of the maximum failure point). In the non-obstacle condition, the DTW values were 9.4 at Level 0, 12.8 at Level 3, and 23.1 at Level 5. In contrast, for the oil condition, which exhibited the highest DTW values, the values were 10.5 at Level 0, 19.1 at Level 3, and 34.5 at Level 5. These experimental results confirm that the body stability of construction workers is influenced by both fatigue levels and external environmental conditions. Further analysis of recovery time, defined as the time it takes for body stability to return to its original level, revealed an increasing trend in recovery time as fatigue levels increased. This study quantitatively demonstrates through wearable sensor data that, as fatigue levels increase, workers experience decreased body stability and longer recovery times. The findings of this study can inform individual worker fatigue management in the future.

## 1. Introduction

Construction workers are vulnerable to falls, slips, and trips (FSTs), as evidenced by the poor accident record in the construction industry [1,2,3,4]. FST accidents occur when a worker loses body stability due to an unstable external environment and fails to regain their lost body stability [5]. In the construction industry, there is substantial evidence of the relationship between loss of body stability and accidents [6]. Since construction workers are often required to work at heights or on unstable surfaces, ensuring body stability is critical to prevent accidents [7]. Workers with poor body stability are more likely to lose their balance and suffer FST accidents, leading to injuries or fatalities [8,9,10,11].

In the construction industry, fatigue can significantly impact body stability. Fatigue can affect posture, balance, and overall stability, making it harder for workers to regain their balance [12,13]. Regarding FST accident occurrence in construction sites, fatigue can negatively influence maintaining and recovering body stability under both non-hazardous and hazardous situations [14,15]. When workers are fatigued, they may experience reduced muscle strength and decreased reaction time. These effects can make it difficult for workers to maintain proper posture and balance [16]. Fatigued workers may have slower and poorer reflexes compared to their non-fatigued state, making it more challenging to react quickly and regain their balance [17,18]. 

According to previous studies, there is clear evidence that fatigue has a negative impact on body stability [19]. Considering that construction workers perform their jobs over extended periods, individual worker fatigue generally accumulates. In other words, the level of fatigue (or magnitude of fatigue) in an individual worker changes over time during a workday. Although the previous studies present evidence of the relationship between the existence of fatigue and body stability [20,21,22], the relationship between levels of fatigue and body stability is not well understood. Furthermore, the relationship between body stability affected by fatigue at different levels and the various environments causing FST accidents at construction sites has also not been fully investigated [23].

Therefore, this study aims to investigate the relationship between different levels of fatigue and body stability and how fatigue affects body stability and recovery under diverse construction site conditions. To analyze the relationship quantitatively, workers’ body stability was measured by data collected from an Inertial Measurement Unit (IMU), which is one of the representative devices for measuring human body movement and stability [24]. Additionally, the different levels of fatigue were applied to workers by utilizing the Havard Step Test (HST). An experiment was conducted in four different environments: non-obstacle, obstacle, and two types of slippery surfaces (water and oil). The data collected in the experiment are analyzed using a time-domain signal processing approach that can represent body stability. This study analyzes the sensor data obtained by IMU through the DTW algorithm and finds the difference between the level of fatigue and body stability in diverse environments. The authors also analyzed the gaits to find the change in recovery. The paper is organized as follows: a literature review covers how to measure fatigue and the application of sensors, followed by detailed explanations of the experimental data and analysis methods in the methodology section. Afterward, the results and discussion are presented.

## 2. Literature Review

In the construction industry, FSTs account for approximately 42.3% of accidents among workers [25]. Previous studies have attributed FST accidents primarily to compromised body stability [26]. Various studies have been conducted to investigate the relationship between body stability and accident occurrence. For instance, Duan et al. [27] investigated the changes in posture and stability of workers during high-altitude work using open pose. Jebelli et al. [28] analyzed the deterioration of body stability due to fall risks by using wearable sensor devices (WIMU) to analyze abnormal walking patterns of workers. Additionally, DiDomenico et al. [29] discovered that the body stability of construction workers acts as a factor causing accidents in high-risk tasks such as falls. 

There have been various studies about diverse factors influencing body stability. Among these studies, Li et al. [30] identified physiological characteristics, personal health, fatigue, age, and physical fitness as contributing factors to decreased body stability and increased accident occurrence. Qu et al. [31] revealed that muscle fatigue, strength, aging, and posture contribute to accidents and decreased balance, examining both internal factors (age, work experience, and fatigue level) and external factors (slippery floors, uneven surfaces, and obstacles) contributing to accidents. These studies commonly emphasize that various factors influence body stability and accident occurrence.

Considering that construction workers mainly perform physical work for extended periods, their fatigue level can potentially affect their body stability [32]. Consequently, studies have been conducted to investigate the relationship between fatigue and body stability from various perspectives [33,34,35,36,37,38]. Escobar-Linero et al. [37] utilized electromyography (EMG) sensors to measure muscle fatigue and evaluated its impact on body stability. Jebelli et al. [39] employed the 3-axis accelerometer embedded in smartphones to detect workers’ gait patterns, identify signs of fatigue, and analyze the relationship between fatigue and body stability. Additionally, Yu et al. [40] collected pressure data from wearable insole devices and used machine learning algorithms to determine and classify the levels of physical fatigue at construction sites. These studies indicate that fatigued workers exhibit greater vulnerability to hazards compared to their non-fatigued counterparts.

One of these advanced sensors, electromyography (EMG), is known to provide a more precise measure of muscle fatigue. EMG sensors can measure muscle activity and fatigue more accurately than traditional methods. However, EMG sensors have several disadvantages, including high cost and difficulty in data collection. Therefore, in this study, we used IMU sensors, which are easier to collect data and more usable, to measure the body stability of workers. However, it is also important to consider that fatigue in construction workers accumulates continuously over time. For instance, a worker’s fatigue level after working for one hour may differ from that after working for seven hours in an eight-hour shift. Even if rest periods are provided at regular intervals, fatigue will accumulate as the working hours increase [41]. To clearly understand how fatigue affects body stability, it is necessary to investigate specifically how various levels of fatigue influence body stability.

Fatigue is a state defined by the inability to sustain the required force for continuous muscle activity or by a decrease in expected efficiency [42]. Physiologically, fatigue is explained by the inability to maintain expected muscle strength or by the weakening of neuromuscular function required for performing tasks and exerting force [43]. Fatigue typically occurs during physical activity [44].

Studies examining the relationship between workers’ fatigue and body stability have mainly focused on measuring accumulated fatigue rather than imposing quantitatively distinct levels of fatigue on workers. For example, Jabelli et al. [45] involve measuring cortisol levels to analyze the impact of fatigue on the walking stability of construction workers. It is necessary to apply various quantifiable levels of fatigue to individual workers. One common method of inducing physical fatigue is by utilizing maximum repetition counts [46]. Lewis et al. [47] measured maximum biceps fatigue by performing arm curls with a 5 kg weight at regular intervals until reaching the maximum repetition count (failure to perform arm curls). Another study confirmed that muscle fatigue reaches its maximum through repeated sprints [48]. A common observation is that as the number of repetitions increases, the level of fatigue also increases. It is generally considered that the fatigue level of the muscles used reaches 100% at the maximum repetition count [49]. Despite efforts to investigate the relationship between fatigue and body stability [50], as well as recovery [51], the clarification of the relationship based on fatigue levels remains insufficient. In this regard, this study aims to investigate the relationship between fatigue levels and body stability and recovery in various construction environments. Table 1 below shows a comparison of key aspects and findings of related studies and our study.

## 3. Methodology

### 3.1. Research Framework

**Figure 1:**
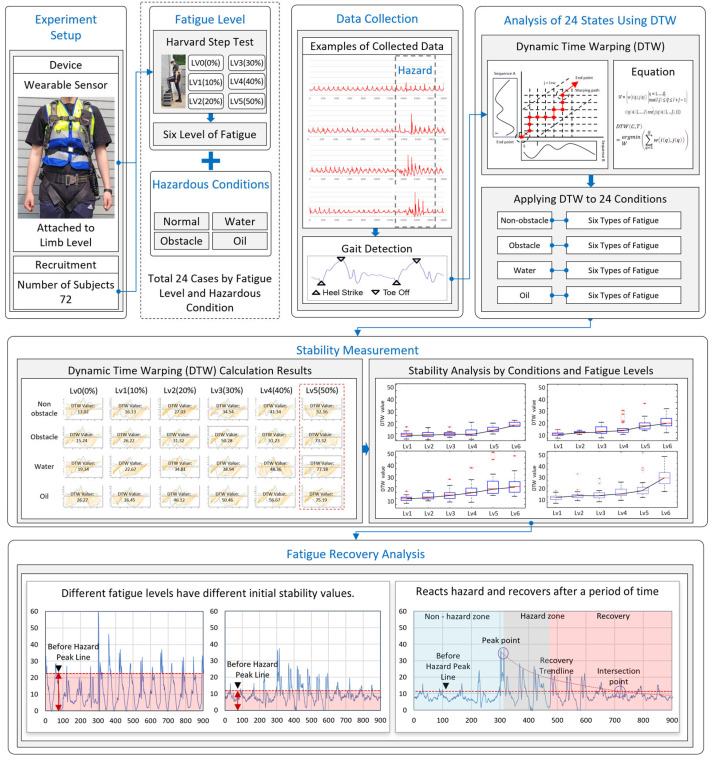
Research framework.

The framework to achieve the research objective is illustrated in Figure 1. First, an IMU sensor was attached to the participant’s waist. Second, to quantitatively measure fatigue, the HST was performed until the failure point, and the maximum repetition count was recorded. In this study, 50% of the maximum repetition count (failure point) was defined as fatigue level 5, 40% as fatigue level 4, and finally, 0% (the fatigue level without performing the HST) was defined as level 0. 

Subsequently, experiments were conducted in four construction environments (non-obstacle, obstacle, water, and oil) for each level of fatigue. In other words, each worker performed a total of 24 experiments (6 fatigue levels and 4 walking environments), with a total of 72 workers. During the experiments, body movements were measured using the IMU sensor. The collected IMU data were processed through a gait detection algorithm [52] in the data collection stage (Figure 1) to classify the gait of each participant. Following that, the DTW algorithm was utilized to determine the workers’ body stability. The measured DTW values were used to analyze body stability in relation to fatigue levels and environmental conditions. 

To address individual differences in body recovery, such as physical condition, underlying diseases, age, and overall health, the authors selected only healthy participants who had no underlying health conditions that would interfere with performing the Harvard Step Test (HST). By selecting healthy participants, the authors aimed to minimize the variability caused by individual health differences and focus on the impact of fatigue and environmental conditions on body stability. All participants underwent a preliminary health screening to ensure they met the necessary health criteria for the study. This approach allowed us to control for potential confounding factors and obtain more reliable results.

To examine the recovery process of body stability, two assumptions were made based on changes in the SVM (signal vector magnitude, defined as level 0) values of the measured IMU data. The first assumption is that the initial stability value may differ depending on the fatigue level. The second assumption is that after workers react to a hazard, their body stability will eventually return to the initial (non-obstacle environment) level after a certain period. By combining these two assumptions, it is possible to analyze the time taken to recover stability by examining the relationships among pre-hazard stability, stability during the hazard, and post-hazard stability, considering the effects of fatigue levels and hazards.

### 3.2. Experimental Design

**Figure 2:**
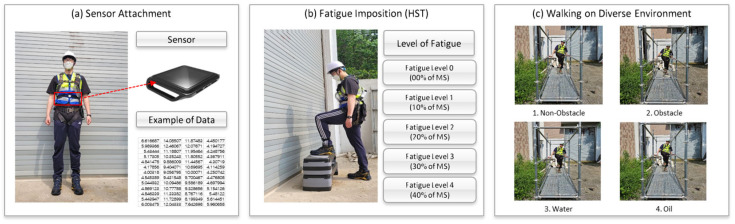
Assessing fatigue levels and body stability on construction sites using IMU sensors and Harvard Step Test: (**a**) sensor attachment; (**b**) fatigue imposition; (**c**) walking in a diverse environment.

In this study, experiments were conducted utilizing IMU sensors to collect data on the impact of fatigue on body stability across various construction environments. To gather the data, an experimental site (total length of 15 m) was constructed using a system scaffold, and four different environmental conditions (non-obstacle, obstacle, water, and oil) were installed to simulate diverse scenarios. During the experiments, participants wore standard personal protective equipment (safety harnesses, safety shoes, and safety helmets) (Figure 2).

**Figure 3:**
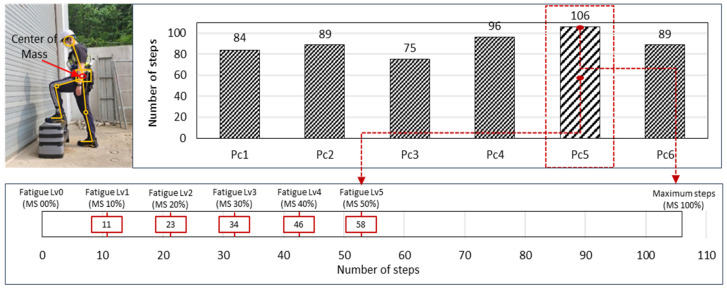
HST failure steps number graph.

The number of repetitions correlates with fatigue levels [53]. Therefore, in this study, measured repetition counts were divided into six levels, from 0% to 50%, assuming a maximum fatigue level of 100%. One of the most suitable tests for measuring fatigue is the HST. The HST evaluates an individual’s aerobic fitness by calculating the ${\mathrm{VO}}_{2}$ max, assessing cardiovascular fitness, and measuring general physical ability, reflecting physical load and recovery capacity [54]. Previous studies have also confirmed a correlation between ${\mathrm{VO}}_{2}$ max and fatigue levels [55], and the HST can be used to measure the threshold for maximum fatigue and artificially control fatigue levels. Figure 3 visually demonstrates the variability in participants’ endurance, showing a clear distribution of failure points across the cohort. This variability highlights the necessity of personalized fatigue level determination, which forms the basis for our subsequent stability analyses under various environmental conditions. 

An IMU sensor was attached to the waist, corresponding to the Center of Body Mass, to measure body stability. Each participant performed the HST at a consistent time interval (120 beats per minute) until they could no longer continue (failure). The failure point is defined as the maximum repetition count they could achieve, and this point is crucial for setting fatigue levels: Level 0 (0%) indicates no fatigue where participants have not performed the HST. Level 1 (10%) to Level 5 (50%) represents incremental fatigue levels set at 10% intervals, up to 50% of the maximum repetition count.

Our choice of up to 50% of the maximum repetition count is based on preliminary studies, which showed that this level induced noticeable fatigue without excessive strain, ensuring participant safety. The increments of 10% were selected to provide a detailed understanding of how different levels of fatigue affect body stability. This approach allows us to quantify fatigue levels in a controlled and consistent manner tailored to each participant’s physical capabilities.

Table 2 shows that the percentage of participants reporting significant fatigue increased notably at the 50% fatigue level, suggesting a threshold at which fatigue becomes more perceivable to the workers. This subjective data complements the objective measurements obtained through the IMU sensors and DTW analysis, providing a comprehensive view of how fatigue levels affect construction workers’ stability and subjective fatigue perception.

The experimental procedure is structured as follows. The participants’ fatigue levels are induced through the HST. Each participant performs one of 24 experiments, with the order of experiments randomly assigned to minimize carryover effects. A one-day rest period is provided after each experiment. For instance, participant A randomly performs one experiment (e.g., walking on an oil surface after 40% of maximum steps (failure point)) out of the 24 randomized experiments, rests for more than one day, and then proceeds to the next randomly selected experiment, completing the entire set of experiments in this manner. To collect tri-axial acceleration data during the experiments, the APDM Opal Movement Monitor was utilized, with data stored at a sampling rate of 128 Hz. The participant information for the 72 participants is presented in Table 3. 

### 3.3. Body Stability Analysis Using the DTW Algorithm

The Dynamic Time Warping (DTW) algorithm is a method for comparing the similarity between two time-series data sets [56]. By considering the time-based misalignment, the similarity between time-series data can be quantified [57]. In this study, the DTW algorithm is employed to compare the participants’ body movements and analyze the changes in stability due to fatigue and working conditions. DTW provides a reliable measure of the similarity between different fatigue levels and body stability patterns, and it is very effective in handling time series data of varying lengths and temporal differences related to fatigue and body stability under different environmental conditions.

First, outliers are filtered from the body movement data collected by the IMU sensors. Subsequently, the DTW algorithm is applied to compare the movement patterns between participants. This allows for the evaluation of changes in participants’ body stability concerning fatigue and working conditions.

In this study, the reference trajectory for computing DTW values was derived from each participant’s gait data during normal walking conditions, specifically, walking without any obstacles and under no fatigue. This baseline condition was selected to reflect the natural, unimpeded walking pattern of each participant. By using everyone’s own non-fatigued, obstacle-free gait as the reference, we aimed to capture deviations in body stability due to varying levels of fatigue and different environmental conditions with high precision.

The DTW algorithm uses local distance measurements to compute a warp path and determine the distance between a reference (class sequence) and a test (test sequence) pattern [58,59]. It is commonly used to analyze the similarity between two time series [60].

Let there be a class sequence ${\left(C\left(i\right)\right)}_{i=1}^{I}$ of length I and a test sequence ${\left(T\left(j\right)\right)}_{j=1}^{J}$ of length *J*, where $C\left(i\right)\in R$ and $T\left(i\right)\in R$. To evaluate the similarity between these two sequences, an *I* × *J* distance matrix *D* is constructed, where $d\left(i,j\right)$ represents the local distance between $C\left(i\right)$ and $T\left(j\right)$. The local distance is typically computed using the squared Euclidean distance, so $d\left(i,j\right)$ can be defined as ${\left(C\left(i\right)-T\left(j\right)\right)}^{2}$. Subsequently, a warping path W is computed from the distance matrix D, representing a set of matrix elements that define mapping and alignment between C and T. W can be expressed as

(1) $$W=\left\{w\left(i\left(q\right),j\left(q\right)\right)\;\left|\begin{array}{c} q=1,\ldots ,Q,\\ \max\left(I,J\right)\leq Q\leq I+J-1 \end{array}\right.\right\},i\left(q\right)\in \left\{1,\ldots ,I\right\}\;and\;j\left(q\right)\in \left\{1,\ldots J\right\}.$$

(2) $$DTW\left(C,T\right)=\begin{array}{c} \arg min\\ W \end{array}\left(\overset{Q}{\underset{q=1}{\sum }}w\left(i\left(q\right),j\left(q\right)\right)\right).\;$$

**Figure 4:**
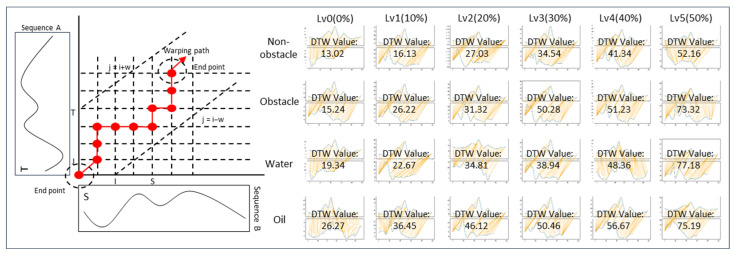
DTW algorithm structure.

Figure 4 illustrates the process of the DTW algorithm and shows examples of DTW values computed under various experimental conditions. The DTW algorithm enables a quantitative comparison of the similarity between the reference gait and the experimental gait.

### 3.4. Body Stability Recovery Based on Fatigue Levels

As mentioned in the Research Framework Section, two assumptions were made to compare the changes and recovery processes of body stability with respect to hazard types and fatigue levels. The two assumptions imply that after responding to an external hazard, workers will recover to their pre-response body stability level. For instance, in the case of no fatigue (fatigue level 1 in this study), the stability before the hazard is maintained until a response to the hazard is observed (decreased stability), after which it recovers to the initial stability level. Combining these two assumptions by examining the relationships among the pre-hazard stability, hazard stability, and post-hazard stability, the recovery process of stability can be assessed based on fatigue levels and hazard types.

**Figure 5:**
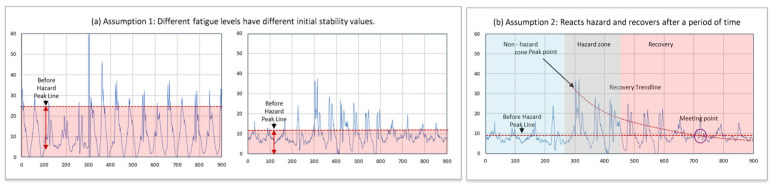
Assessing body stability recovery time: an examination of pre-hazard, hazard, and post-hazard stability dynamics. (**a**) Assumption 1: different fatigue levels have different initial stability values. (**b**) Assumption 2: reacts to hazard and recovers after a period.

Figure 5 illustrates an example of the assumptions. It depicts the body stability recovery time for a specific participant under different fatigue levels. This graph examines the relationship between recovery time and hazard types as well as fatigue levels. The graph includes “Peak point” and “Meeting point” labels. The “Peak point” represents the lowest individual stability, exhibiting the highest DTW value. In contrast, the “Meeting point” indicates the point where the individual’s fatigue level begins to return to the baseline, marking the start of stability recovery. The distance between these two points represents the time required for the individual to regain body stability.

## 4. Result

### 4.1. Investigation of Body Stability Changes Due to Fatigue in Various Construction Environments Using DTW Analysis 

**Figure 6:**
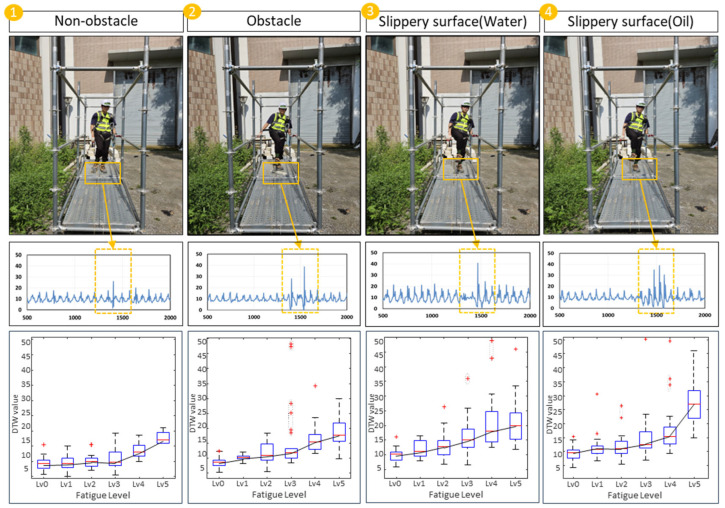
Investigation of body stability changes due to fatigue in various construction environments using DTW analysis.

The investigation of body stability changes across 24 cases using DTW yielded the following results. The DTW values derived from the experimental data are presented in Figure 4, classified according to different external environments and fatigue levels. In this study, the DTW values are calculated based on the gait data at the hazard installation point rather than using all gait data throughout the entire experiment. In other words, the DTW values are computed from the data represented by the orange box in the gait data shown in Figure 6.

For non-obstacle (A), the average DTW value during the non-fatigued state was 8.0. However, as fatigue accumulated through the HST, corresponding to fatigue levels 1 (10%), 2 (20%), and 3 (30%), the DTW values were 9.4, 9.7, and 10.7, respectively. The changes in DTW values due to fatigue were relatively subtle during the initial level 0–3 (0–30%) intervals, with more significant differences observed between the level 4–5 (40–50%) intervals compared to the level 0–3 (0–30%) intervals. In the obstacle (B) case, the average DTW value was 9.5. Similarly, with fatigue accumulation due to HST, the DTW values for fatigue levels 1 (10%), 2 (20%), and 3 (30%) were 11.2, 12.0, and 15.3, respectively. Like the non-obstacle environment, the effect of fatigue was more pronounced at the level 4–5 (40–50%) intervals. At fatigue levels 4 (40%) and 5 (50%), the DTW values increased to 18.9 and 24.8, respectively. For Water (C), the average DTW value was 9.7. As the fatigue level increased from level 1 to level 3 (30%), the corresponding DTW values were 10.3, 13.0, and 15.9, respectively. At fatigue levels 4 (40%) and 5 (50%), the DTW values were 18.9 and 26.7, exhibiting a linear change even at higher fatigue levels, conditions (non-obstacle (A), obstacle (B), and oil (D)). In the case of oil (D), the average DTW value without fatigue was 10.5. At fatigue levels 1 (10%), 2 (20%), and 3 (30%), the DTW values increased to 15.5, 16.3, and 19.1, respectively, with the highest values observed at levels 4 and 5, being 20.1 and 34.5, respectively.

As the fatigue level increases, the body stability (DTW value) in response to external environments or hazards tends to decrease. Additionally, the external environment itself was found to have a significant impact on body stability. A comparison across environments reveals that the following trends for non-obstacle (A) and obstacle (B) differ, but the value ranges are similar. When comparing obstacle (B) and slippery surface (water (C)), the values are higher than obstacle (B), but the trends are similar. Comparing slippery surface (water (C)) and slippery surface (oil (D)), the ranges are similar, but the values are higher for slippery surface (oil (D)). Particularly, when conducting the HST at level 4 (40%) or higher, the DTW values significantly increase, reaching 18.9 (a 42% increase compared to fatigue level 1) for obstacle (B) and 26.7 (a 133% increase compared to fatigue level 1) for slippery surface (water (C)). Despite the changes in external environmental conditions, obstacle (B) and slippery surface (Water (C)) exhibit similar DTW values. For slippery surface (oil (D)), the DTW values in the range of levels 1–3 (10–30%) were 15.5, 16.3, and 19.1, respectively, higher than the average values observed in conditions (A), (B), and (C). When the repetition exceeded level 4 (40%), the DTW values increased to 18.9 and 24.8, representing a 61.0% increase from level 0 (0%) to level 3 (30%). 

The most prominent difference is observed between the non-obstacle (A) environment and slippery surface (oil (D)), where body stability decreases significantly with increasing fatigue in condition (D). In (A), the DTW value at level 5 (50%) is approximately 23.1, whereas in (D), the average DTW value when fatigue level 5 (50%) is imposed is 34.5. This implies that workers experiencing decreased body stability due to fatigue may exhibit further reductions in body stability in hazardous situations. In other words, increased fatigue levels could make it more difficult to effectively respond to hazards. Specifically, in condition (D), the significantly lower surface friction coefficient makes it challenging for workers to maintain balance, resulting in a rapid increase in DTW values.

Consequently, analyzing the correlation between DTW values and fatigue levels demonstrates that fatigue levels are inversely related to body stability. Examining the Pearson correlation values for each environment reveals that the correlations between fatigue levels and DTW are (A) 0.996, (B) 0.986, (C) 0.977, and (D) 0.989, respectively.

### 4.2. Results of Body Stability Recovery Analysis for Six Fatigue Levels

**Figure 7:**
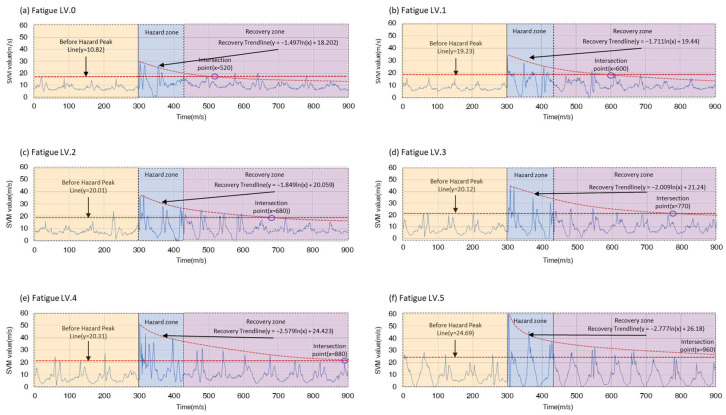
Recovery analysis graph for six different fatigue levels: (**a**) fatigue level 0 (0%), (**b**) fatigue level 1 (10%), (**c**) fatigue level 2 (20%), (**d**) fatigue level 3 (40%), (**e**) fatigue level 4 (40%), (**f**) fatigue level 5 (50%).

Figure 7 illustrates an example of the changes in recovery across individual participants’ fatigue levels in the fatigue recovery analysis. Starting from fatigue level 0 (0%), the “Before Hazard Peak Average Line” is set at y = 10.82, and accordingly, the peak value within the “hazard zone” is 30, indicating that fatigue begins at this peak point. As fatigue decreases, the recovery trendline follows the equation y = −1.497ln(x) + 18.202. The intersection of this recovery curve with the threshold y = 10.82 represents the recovery interval, calculated at x = 520, indicating the point where gait stability returns to the pre-hazard zone level. As the fatigue increases from level 0 (0%) to level 1 (10%), the y-value rises from 10.82 to 19.23 in correspondence with the overall DTW values. The peak value within the “Hazard Zone” is observed as 32. The recovery trendline is given by y = −1.711ln(x) + 19.44. This recovery intersection point is calculated at x = 600. When the fatigue level increases to 3, the average value of the “Before Hazard Peak Average Line” is y = 20.12. A peak value is observed within the “Hazard Zone”, and the recovery trendline is defined as y = −2.009ln(x) + 21.24. This recovery curve indicates the point where body stability recovers as fatigue decreases. The recovery point is calculated at x = 770. Finally, when the fatigue level increases to 5, the analysis of the “Before Hazard Peak Average Line” yields an average value of y = 24.69. A peak value exists within the “Hazard Zone”, indicating an increase in fatigue. The recovery trendline is modeled as y = −2.777ln(x) + 26.18, and this recovery curve represents the point where body stability recovers as fatigue decreases. This recovery point occurs at x = 960.

As the fatigue level increases from 0 (0%) to 5 (50%), the “Before Hazard Peak Average Line” continuously rises, starting from 10.82 at level 0 and reaching 24.69 at level 5. This increasing trend indicates an increase in physical burden as the fatigue level increases. Additionally, the maximum fatigue value is 30 at 0% fatigue level and increases to 62 at level 5 (50%). This increase in peak values emphasizes the increase in physical load over time. The recovery trendlines are expressed as logarithmic functions of the form y = −a ln(x) + b. Notably, the recovery rate increases as the fatigue level increases, which is due to the increase in the coefficient ‘a’ in the logarithmic equation.

The most significant change is that the values increase rapidly when the fatigue level reaches level 5 (50%). For levels above 5, this indicates a substantial increase in physical load and the time required for recovery, which can negatively impact worker performance and stability. This tendency is evident from the observation that the ‘x’ value of the intersection point increases as the fatigue level rises, suggesting a requirement for a longer recovery time for the body to regain body stability.

### 4.3. Comparative Analysis of Fatigue Levels and Working Environment Conditions on Overall Worker Stability and Recovery Time

**Figure 8:**
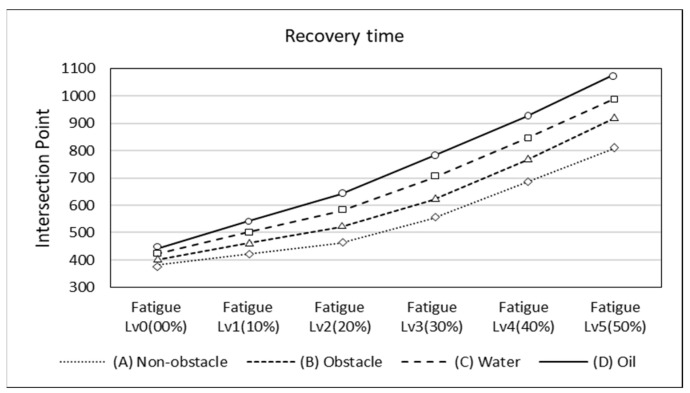
Graph of changes in worker stability and responses based on fatigue levels and hazard types.

Figure 8 compares the recovery time of overall worker body stability under various working environment conditions and fatigue levels. The recovery time was measured by the X-coordinate value of the intersection points, and the fatigue level was classified into six stages from Level 0 (0%) to Level 5 (50%). The working environment conditions were categorized into four types: non-obstacle (A), obstacle (B), water (C), and oil (D).

According to the results, as the fatigue level increased from Level 0 (0%) to Level 5 (50%), the X-coordinate value, representing the recovery time, continuously increased. In general, the higher the fatigue level, the greater the increase in recovery time, with the highest increase rate observed at the Level 5 (50%) fatigue level. Among the working environment conditions, the X-value was highest in the oil (D) condition and lowest in the non-obstacle (A) condition. In all conditions, the recovery time increased as the fatigue level rose, with the largest increase rate observed from fatigue Level 4 (40%) to Level 5 (50%).

Furthermore, at the same fatigue level, differences in recovery time existed depending on the working environment condition. The lowest X-value was recorded in the non-obstacle (A) condition, while the highest X-value was observed in the oil (D) condition. With regards to the above, the results suggest that, as the fatigue level increases, adverse working conditions may have a greater impact on working performance.

## 5. Discussion

### 5.1. Changes in Body Stability According to Fatigue Levels and Hazard Types

The findings regarding the relationship between fatigue levels and hazard types provide an important foundation for exploring the association between work environment stability and individual responses. According to the experimental results, individuals exhibit varying responses to different hazard types as their fatigue levels increase. These results are inferred to be due to the physical characteristics of the hazards and individual differences in fatigue responses. For instance, one worker A exhibited a rapid increase in fatigue starting from level 3 (30%), whereas other workers did not show a significant increase in fatigue even after levels 1 (10%) or 2 (20%). These results indicate that when dividing fatigue levels from 0 (0%) to 5 (50%), worker A experienced a more substantial decrease in stability at higher fatigue levels compared to other workers. Particularly, in the same environment, worker A exhibited a distinct decrease in body stability after level 3 (30%), while other workers showed a response after level 2 (20%). This suggests that there are individual differences in response times to fatigue among workers. Such variations indicate that individual responses to fatigue can differ. Consequently, it is necessary to measure each worker’s fatigue level and provide appropriate rest schedules.

Furthermore, the analysis of stability changes across different hazard types revealed that the physical characteristics of the environment interact differently with worker fatigue. Notably, hazard types such as obstacles or slippery surfaces appeared to be more sensitive to worker fatigue. Taking the example of working on a typical slippery surface in construction sites such as a urethane floor in a bathroom, two cases can be considered. Firstly, the urethane floor may lead to a decrease in body stability due to fatigue, and the recovery time may be prolonged. To mitigate this risk, workers should be provided with adequate rest, and preventive measures should be taken to address fatigue-induced stability reductions. Secondly, the urethane floor surface may exhibit relatively high stability levels when fatigue is low but lower stability when fatigue is high. This implies that for workers with low fatigue levels, the urethane floor surface may not pose a hazard, but it could become a risk factor for slips or falls when fatigue levels are high. In such cases, workers should be provided with sufficient rest, or the hazard should be eliminated before fatigue levels reach a point where stability is compromised to the extent of causing accidents.

**Figure 9:**
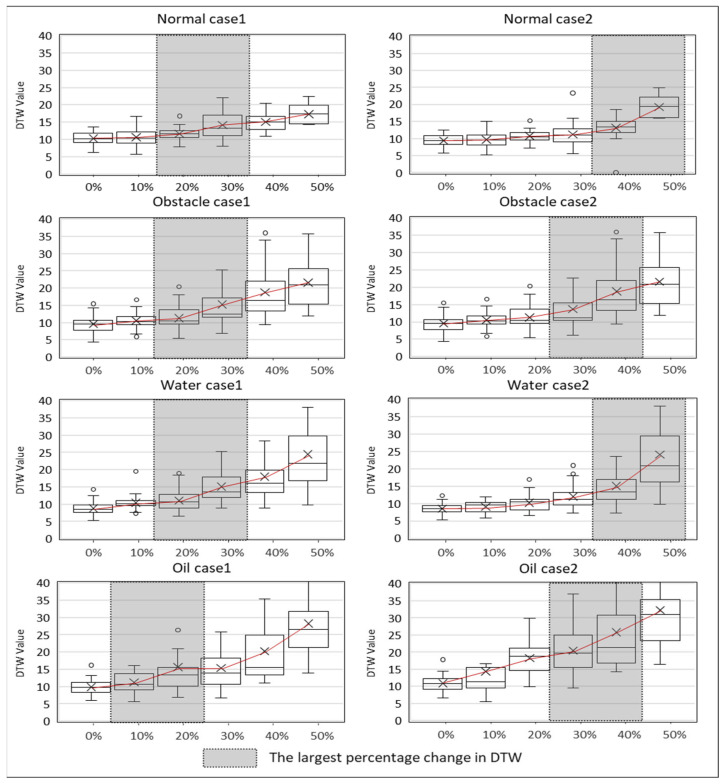
Stability Changes with Increasing Fatigue Across Various Environments.

Figure 9 presents a comprehensive analysis of how worker stability changes in response to various fatigue levels and hazard types. The figure includes multiple sub-graphs, each depicting the relationship between DTW values (indicative of body stability) and different fatigue levels (0%, 10%, 20%, 30%, 40%, and 50%) across various environmental conditions.

Non-obstacle (A) shows that DTW values increase moderately as fatigue levels rise, indicating a gradual decline in stability. For instance, DTW values increased from 8.0 at 0% fatigue to 23.1 at 50% fatigue, showing a gradual but steady increase. Obstacle (B) is DTW values show a more significant increase at higher fatigue levels, reflecting the added complexity of navigating obstacles. The values ranged from 9.5 at 0% fatigue to 24.8 at 50% fatigue, demonstrating a sharper increase compared to the non-obstacle environment. Water surface (C) DTW values exhibit a linear increase with rising fatigue levels, emphasizing the additional challenge posed by slippery conditions. DTW values rose from 9.7 at 0% fatigue to 26.7 at 50% fatigue, indicating the significant impact of the water surface on stability as fatigue increases. Oil surface (D) demonstrates the highest DTW values at each fatigue level, highlighting the severe impact of low friction surfaces on stability, especially under fatigue. DTW values increased from 10.5 at 0% fatigue to 34.5 at 50% fatigue, showing a substantial decrease in stability on the oil surface. These detailed sub-graphs collectively underscore the interaction between fatigue and environmental hazards, showing that higher fatigue levels generally correlate with increased DTW values, hence decreased stability.

### 5.2. Impact of Fatigue Levels and Hazard Proximity on Worker Body Stability in Construction Environments

Additional case tests were conducted to assess the importance of maintaining a certain level of stability (low DTW values) in work environments. The purpose of the case tests was to examine (1) how the range variability of body stability changes when workers encounter consecutive hazards in a state of reduced stability and (2), based on this, to confirm the need to manage worker fatigue levels within a certain range.

The tests were divided into four cases: (a) sparsely positioned Hazard Zones at fatigue level 3 (30%), (b) densely positioned Hazard Zones at fatigue level 3 (30%), (c) sparsely positioned Hazard Zones at fatigue level 5 (50%), and (d) densely positioned Hazard Zones at fatigue level 5 (50%).

**Figure 10:**
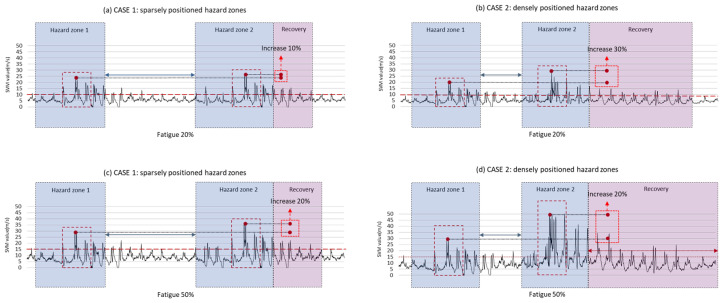
Impact of fatigue levels and hazard proximity on worker body stability in construction environments: (**a**) fatigue 20% sparsely positioned hazard zones; (**b**) fatigue 20% densely positioned hazard zones; (**c**) fatigue 50% sparsely positioned hazard zones; (**d**) fatigue 50% densely positioned hazard zones.

Figure 10 presents the test results. The test results confirmed that changes in stability, expressed by DTW values, occur in response to consecutive hazards, depending on the fatigue level. When the hazard zones are sparsely positioned, and the fatigue level is 3 (30%), the DTW value increases by approximately 10%, from 27.98 at the first hazard to 30.56 at the second hazard (see Figure 10a). In contrast, when the fatigue level is 5 (50%), the DTW value increases by 30%, from 25.64 at the first hazard to 37.64 at the second hazard (see Figure 10b). This trend is more pronounced in densely positioned hazard zones.

In densely positioned hazard zones, when the fatigue level is 3 (30%), the DTW value increases by 20%, from 28.39 at the first hazard to 37.47 at the second hazard (see Figure 10c). However, when the fatigue level is 5 (50%), the DTW value increases by 50%, from 29.98 at the first hazard to 56.52 at the second hazard (see Figure 10d). In other words, stability in spaces where hazards are scattered can be influenced by the worker’s fatigue level.

A notable aspect of the case tests conducted in this study is the relationship between the spacing of successive hazard zones and fatigue levels. In the sparsely spaced hazard zones, workers faced the second hazard only after they had recovered a certain level of stability after the first hazard. In this case, the increase in DTW value due to the first hazard was sufficiently recovered before facing the second hazard, and the impact of the second hazard on physical stability was lower than the increase in the dense hazard zones. In contrast, in the dense hazard zones, it was difficult for workers to maintain stability in the face of successive hazards, as changes in stability occurred quickly, and workers often faced the next hazard before recovering.

These results emphasize that both fatigue levels and hazard spacing are crucial factors in managing stability and fatigue in work environments. The higher the fatigue level and the narrower the hazard spacing, the slower the recovery of body stability, increasing the likelihood of more significant accidents. Therefore, efficient work schedules and safety policies should be considered to improve worker safety and performance, taking these factors into account.

### 5.3. Contributions and Limitations

This study analyzed the relationships among fatigue levels, hazard types, and body stability through various experiments. The experimental results revealed that as fatigue levels increase, the impact on hazards becomes more significant, particularly in the oil (D) environment, where body stability decreases substantially. This relationship highlights the importance of managing fatigue and hazards to maintain safety in work environments. This will help identify and improve factors affecting worker safety in specific processes and work environments, predict changes in stability due to fatigue, and prevent accidents.

Through the recovery analysis for six fatigue levels, it was found that the rate of stability recovery decreases as the fatigue level increases. Notably, the recovery rate drops rapidly when the fatigue level reaches 50%. These results indicate the need for effective stability recovery strategies in fatigued states, which is particularly crucial at high fatigue levels. This information can be utilized as a reference for adjusting work schedules and work environments based on workers’ fatigue states. 

The research findings provide a theoretical basis for applying rest periods based on fatigue levels in field safety management. Considering the relationship between fatigue and hazards, it is necessary to carefully monitor workers’ fatigue states and allow for more frequent breaks in cases of high fatigue levels and specific work environments. This is expected to provide important guidance for developing future fatigue management strategies to optimize worker safety and work efficiency.

This study is limited by several aspects. Firstly, the experiments were conducted on simulated work platforms. While the relationships among fatigue levels, body stability, and hazards were identified through experiments under specific conditions, further investigation is needed to determine the applicability of the results to various real construction site situations. Secondly, to account for individual differences and diverse physical characteristics of the experimental participants, many construction workers were recruited. However, to develop and apply a general fatigue management model, continuous data collection from more participants is necessary. This will enable the creation of a universally applicable model and system for managing worker fatigue. Finally, factors other than fatigue may influence worker body stability. For instance, work environment, work intensity, individual health conditions, and other factors may interact in a complex manner. Future research should adopt a more comprehensive approach by considering these additional factors.

### 5.4. Future Research

This study primarily focused on the impact of fatigue on lower body stability due to the nature of the Harvard Step Test (HST), which targets lower body movements. While our findings provide valuable insights into the relationship between lower body fatigue and body stability, they are limited in their generalizability to other types of fatigue.

Future research should incorporate tasks that simulate upper body exertion to provide a more comprehensive understanding of the relationship between fatigue and body stability. For instance, activities such as carrying varying weights while walking on different surfaces can mimic common construction tasks that involve significant upper-body effort. This will help in assessing the impact of upper-body fatigue on overall body stability.

Additionally, integrating more complex and diverse tasks could enhance the practical applicability of our findings to various construction activities. Future studies should design experiments that include both upper and lower body tasks and use advanced wearable sensors to capture comprehensive movement data. This approach will help better understand the multifaceted nature of fatigue and its effects on construction workers’ safety and performance.

By addressing both lower and upper-body fatigue, future research will provide a more holistic view of the relationship between fatigue and physical stability, which could lead to more effective fatigue management strategies in the construction industry.

## 6. Conclusions

Many construction worker accidents stem from FSTs, which are often directly related to loss of body stability. The harsh environments and continuous physical labor on construction sites easily lead workers to fatigue, which undermines body stability and increases the risk of accidents. Especially in construction sites with various hazards such as obstacles, water, and oil, maintaining worker body stability is essential. Therefore, this study focused on investigating the relationship between worker fatigue and body stability in various construction environments. Specifically, we analyzed workers’ fatigue levels and DTW values in different construction environment conditions (non-obstacle, obstacle, water, oil) to identify factors affecting worker safety and performance.

To assess the relationship between worker fatigue levels and body stability, a comprehensive experiment related to body stability, fatigue, and recovery was conducted. During the experiments, workers wore IMU sensors attached to their waist to track movements and postures in real time, and the collected data were processed using DTW. By comparing and analyzing workers’ movement patterns, we evaluated the relationship between fatigue and body stability. 

The experimental results demonstrated a significant increase in DTW values as fatigue levels increased. Specifically, under non-obstacle conditions, the increase in DTW values was more gradual at lower fatigue levels but showed a pronounced jump at higher fatigue levels, indicating critical thresholds where stability deteriorates significantly. This indicates that body stability changes progressively from fatigue levels 1 to 5, with critical thresholds at higher levels.

Moreover, the authors explored how different environmental conditions, such as non-obstacle, obstacle, water, and oil surfaces, affect body stability at each fatigue level. This analysis revealed that slippery surfaces like oil exacerbate the impact of fatigue more significantly, even at lower fatigue levels, compared to non-slippery surfaces. This highlights the necessity of addressing environmental conditions in fatigue management strategies.

A detailed analysis of recovery times also showed that higher fatigue levels not only resulted in longer recovery times but also changed the dynamics of recovery. The analysis of recovery time as a function of fatigue level showed that the time required for recovery increased significantly as fatigue level increased, especially beyond fatigue level 5. These results emphasize the importance of managing workers’ fatigue and ensuring adequate rest periods.

## Figures and Tables

**Table 1:** Comparison of key aspects and findings of related studies and our study.

Study	Methodology	Sensor Types Used	Environmental Conditions Considered	Fatigue Assessment Method	Key Findings
Duan et al. (2021) [31]	work modeling of unsafe behaviors	Open pose camera system	High-altitude work	Not specified	Analyzed changes in posture and stability during high-altitude work.
Ebell et al. (2016) [32]	Fall risk analysis using wearable sensors	Wearable IMU devices	Construction site	Gait pattern analysis	Found that fall risks can be assessed using postural stability metrics from wearable sensors.
Li et al. (2020) [33]	Mental fatigue classification using eye-tracking	Wearable eye-tracking device	Simulated construction environment	Eye-tracking fatigue assessment	Classified mental fatigue of construction equipment operators using eye-tracking technology.
Yu et al. (2019) [34]	Physical workload estimation	Wearable insole pressure system	Construction site	Machine learning algorithms	Developed methods to estimate physical workload using computer vision and insole pressure data.
Escobar-Linero et al. (2022) [35]	Physical fatigue classification using neural networks	EMG sensors	Laboratory	Neural network analysis	Used neural networks to classify worker physical fatigue based on EMG sensor data.
Chan (2011) [36]	Analysis of fatigue as a critical accident risk	Not specified	Oil and gas construction sites	Observation and interviews	Identified fatigue as a critical factor contributing to accidents in the oil and gas construction sector.
This Study (2024)	Body stability analysis using DTW	IMU sensors	Non-obstacle, obstacle, water, oil	HST: until failure, with fatigue levels set at 10–50%	Demonstrated significant increases in DTW values as fatigue levels increased and identified the importance of fatigue management.

**Table 2:** Participants reporting significant fatigue at various fatigue levels.

Fatigue Level (%)	Participants Reporting Significant Fatigue	Total Participants	Percentage Reporting Significant Fatigue (%)
30	5	72	6.94
40	15	72	20.83
50	28	72	38.89
60	13	72	18.06
70	11	72	15.28

**Table 3:** Subject information.

	Height (cm)	Weight (kg)	Age (Years)
Mean	175.67	74.95	54.23
Median	173.74	67.28	52.01
Standard Deviation	6.54	15.21	3.21
Min Value	163.45	56.98	45.11
Max Value	178.7	87.35	58.23

## Data Availability

All data, models, or code generated or used during the study are available from the corresponding author by request.
